# Proteomic analysis of pitcher fluid from *Nepenthes* × *ventrata*

**DOI:** 10.1016/j.dib.2018.01.037

**Published:** 2018-02-02

**Authors:** Wan Nor Adibah Wan Zakaria, Wan Mohd Aizat, Hoe-Han Goh, Normah Mohd Noor

**Affiliations:** Institute of Systems Biology, Universiti Kebangsaan Malaysia, UKM Bangi, 43600 Selangor Darul Ehsan, Malaysia

**Keywords:** Carnivorous plant, Digestive enzyme, Nepenthes, Proteomics, SWATH

## Abstract

The carnivorous plants of genus *Nepenthes* produce unique pitchers containing secretory glands, which secrete proteins into the digestive fluid. We investigated protein profile in the pitcher fluid during the first three days of opening to understand carnivory trait of *Nepenthes* × *ventrata*. The proteome analysis of pitcher fluid from *N.* × *ventrata* was performed by label-free quantitative liquid chromatography mass spectrometry (nLC-MS/MS^ALL^). Raw MS data have been deposited to the ProteomeXchange with identifier PXD007251. This dataset allows the identification and quantification of proteins from pitcher fluids to elucidate proteins involved in carnivory physiology of *Nepenthes* species.

**Specifications**

**Table:** 

Subject area	Biology, Plant Molecular Biology
Specific subject area	Proteomics
Type of data	LC-MS spectral data
Organism/Cell line/tissue	*Nepenthes* × *ventrata* (Pitcher fluid)
How data was acquired	MS analysis using TripleTOF 5600 (AB Sciex)
Data format	Raw (.wiff &.scan)
Experimental features	Label-free quantitative nLC-MS/MS^ALL^ proteomic analysis of pitcher fluid from *N.* × *ventrata*
Sample source location	Bangi, Malaysia (2°55′11.5′′N 101°47′01.4′′E)
Data accessibility	Data are available via ProteomeXchange with identifier PXD007251 (http://www.ebi.ac.uk/pride/archive/projects/PXD007251)

**Direct link to deposited data**

http://www.ebi.ac.uk/pride/archive/projects/PXD007251.

**Value of the data**

• This is the first quantitative proteomic dataset of a carnivorous plant species.
• It can be used to identify and quantify proteins from the digestive fluid of *N*. × *ventrata* from newly opened pitcher and pitcher after three days of opening.
• Protein identification from pitcher fluid during early days of pitcher opening is important to understand the endogenous protein secretion system, prior to influence of external factors, such as prey induction and environmental stimuli.
• Proteins in pitcher fluid are in very low concentrations, which posed a challenge for comprehensive detection and identification of peptide signals from mass spectrometry (MS) analysis.
• Combination of this proteomic dataset with Nepenthes transcriptomes [2], [3], [4] will allow the discovery and expression study of digestive enzymes and other proteins which are secreted into the pitcher fluid.

## 1 Data

In this dataset, we present proteomic profiling of proteins with size > 10 kDa from pitcher fluid of *N.* × *ventrata.* Two sets of data were deposited; one is for fluid samples from newly opened pitcher, while another one is from longevity experiment of pitchers after three days of opening. Three replicates of each sample were pooled together into one MS run to ensure better signal detection. The mass spectrometry proteomics data of three technical replicates each were deposited to the ProteomeXchange Consortium (http://proteomecentral.proteomexchange.org) via the PRIDE [5] partner repository, with the dataset identifier PXD007251. These include i) LC-MS/MS^ALL^ raw data (.wiff &.scan), ii) ion library search result (.txt), and iii) reference transcriptome of predicted protein sequences (.fasta).

## 2 Experimental design, materials and methods

### 2.1 Plant materials

*Nepenthes* × *ventrata* plants were grown under shady environment in experimental plot (2°55′09.0′′N 101°47′04.8′′E) at Universiti Kebangsaan Malaysia, Bangi. Two different set of pitchers were collected as samples. Control set consists of pitcher fluids collected within 24 h of pitcher opening. For pitcher fluid study, collected pitcher fluid samples were processed according to [1]. For longevity experiment, emptied pitchers from control set were replenished with the filtrate depleted of proteins larger than 10 kDa and sealed with parafilm for 3 days before sample collection. Presence of proteins from the pitcher fluid were visualized with SDS-PAGE prior to LC-MS analysis.

### 2.2 Solid phase extraction and trypsin digestion

Proteins from pitcher fluid were separated from other impurities using commercially available 1 cc SPE cartridges with Waters Oasis HLB sorbent (Waters, USA), followed by reduction with DTT and alkylation with IAA before digestion with trypsin. Digestion was done overnight at 37 °C. Three biological replicates were pooled for LC-MS detection.

### 2.3 Mass spectrometry and data analysis

TripleTOF 5600 system (AB SCIEX, Framingham, MA) coupled with an Eksigent NanoLC-ultra 2D+ with Nanoflex cHiPLC system (AB SCIEX) were used for LC-MS/MS analysis. Samples were loaded into a Eksigent nano cHiPLC Trap column at a flow rate of 3 µL/min for 10 min and resolved on a nano cHiPLC analytical column with a linear gradient of solvent B (98% ACN containing 0.1% FA) at a flow rate of 300 nL/min. The nanoLC column was rinsed with 90% solvent B equilibrated with 95% solvent A. LC-MS/MS runs were performed in triplicates per sample. All spectra generated from IDA acquisitions were searched against the predicted peptide database from *N*. × *ventrata* reference transcriptome [2] using ProteinPilot™ v4.5 (AB SCIEX) for protein and peptide identifications. Peptide detected was filtered by false discovery rate, FDR < 0.001.
